# Onchocerciasis-associated epilepsy: an explorative case-control study with viral metagenomic analyses on** *Onchocerca volvulus*

**DOI:** 10.12688/f1000research.138774.1

**Published:** 2023-10-04

**Authors:** Amber Hadermann, Stephen Raimon Jada, Wilson J. Sebit, Thomas Deng, Yak Y. Bol, Joseph N. Siewe Fodjo, Lander De Coninck, Jelle Matthijnssens, Inge Mertens, Katja Polman, Robert Colebunders

**Affiliations:** 1 Global Health Institute, University of Antwerp, Antwerp, 2610, Belgium; 2 Amref Health Africa, Juba, South Sudan; 3 Public Health Laboratory, Ministry of Health South Sudan, Juba, South Sudan; 4 Maridi Hospital, Maridi, South Sudan; 5 Neglected Tropical Diseases Unit, Ministry of Health South Sudan, Juba, South Sudan; 6 Laboratory of Viral Metagenomics, KU Leuven, Leuven, Flanders, 3000, Belgium; 7 Health Unit, VITO (Vlaamse Instelling voor Technologisch Onderzoek), Mol, 2400, Belgium; 8 Centre for Proteomics, University of Atwerp, Antwerp, Belgium; 9 Department of Health Sciences, VU Amsterdam, Amsterdam, 1081, The Netherlands; 10 Department Public Health, Institute of Tropical Medicine, Antwerp, 2600, Belgium

**Keywords:** Onchocerciasis, epilepsy, nodulectomy, pathogenesis, metagenomics, Onchocerca volvulus, virome

## Abstract

**Background:** A high prevalence of onchocerciasis-associated epilepsy (OAE) has been observed in onchocerciasis-endemic areas with high ongoing *Onchocerca volvulus* transmission. However, the pathogenesis of OAE remains to be elucidated. We hypothesise that the *O. volvulus* virome could be involved in inducing epilepsy. With this study, we aim to describe the *O. volvulus* virome and identify potential neurotropic viruses linked to OAE.

**Methods:** In Maridi County, an onchocerciasis endemic area in South Sudan with a high prevalence of OAE, we will conduct an exploratory case-control study enrolling 40 persons aged 12 years and above with palpable onchocerciasis nodules. Cases will be participants with OAE (n=20), who will be age- and village-matched with controls without epilepsy (n=20). For each study participant, two skin snips at the iliac crest will be obtained to collect *O. volvulus* microfilariae, and one nodulectomy will be performed to obtain adult worms. A viral metagenomic study will be conducted on microfilariae and adult worms, and the *O. volvulus* virome of persons with and without OAE will be compared. The number, size, and localisation of onchocerciasis nodules in persons with and without OAE will be described. Moreover, the pre- and post-nodulectomy frequency of seizures in persons with OAE will be compared.

**Ethics and dissemination:** The protocol has been approved by the Ethics Committee of the University of Antwerp and the Ministry of Health of South Sudan. Findings will be disseminated nationally and internationally via meetings and peer-reviewed publications.

**Registration:** https://clinicaltrials.gov/ registration NCT05868551 ( https://clinicaltrials.gov/study/NCT05868551)

**Protocol version**: 1.1, dated 09/05/2023

## Introduction

*Onchocerca volvulus* is a filarial nematode parasite transmitted by blackflies ( *Simulium*), which causes onchocerciasis, commonly referred to as “River Blindness.” Recent epidemiological studies strongly suggest that onchocerciasis may directly or indirectly induce epilepsy, known as “Onchocerciasis-associated epilepsy” (OAE). ^1^^,^^2^ Onchocerciasis-associated epilepsy appears in previously healthy children between the ages of 3–18 years, with a peak onset at 8–11 years, ^1^ and comprises a broad spectrum of seizures, including generalised tonic-clonic, absence and nodding seizures, the latter as part of nodding syndrome. ^3^^,^^4^ Some OAE cases may also have stunting with delayed puberty, as part of Nakalanga syndrome. ^1^ Until now, diagnosing OAE has been difficult. An OAE case definition for epidemiological studies has been proposed, ^5^ but there is currently no test to confirm that epilepsy in a child is caused by onchocerciasis. Moreover, the fact that the pathogenesis of OAE remains unknown has kept this important public health problem from being internationally acknowledged and made it challenging to direct the necessary resources to the affected communities which are often very remotely located. ^5^

Multiple hypotheses concerning the pathogenesis of OAE are currently being explored. ^6^ It has been suggested that *Wolbachia*, an intracellular symbiotic microorganism critical for the survival of the *O. volvulus* parasite, could be involved, as *Wolbachia* has been linked to the disease mechanism of onchocerciasis-related blindness. ^7^ However, recent studies did not detect *Wolbachia* DNA in cerebrospinal fluid and post-mortem brain tissue of persons with OAE. ^8^

To our knowledge, the virome of *O. volvulus* has never been investigated. In other parasites, a wide range of RNA viruses have been identified. For some of these parasite-hosted viruses, such as the rhabdoviruses found in *Schistosoma solidus* and a series of nematodes, it has been suggested that they may contribute to the infection dynamics. ^9^^–^^12^ Rhabdoviruses are negative-sense single-stranded RNA viruses known to infect a range of different hosts, from mammals, including humans, to arthropods, plants, and parasites. ^13^ The fact that these viruses have such a wide range of hosts raises the question of whether these viruses are impacting multiple species by being able to infect multiple hosts naturally and/or through evolution by host jumping. We hypothesise that the *O. volvulus* virome could be involved in the pathogenesis of OAE.

In a pilot exploratory viral metagenomic study, we performed virus-like particle (VLP) enrichment according to the NetoVIR protocol, ^14^ followed by Illumina deep sequencing on *O. volvulus* adult worms obtained from persons without epilepsy in Cameroon (62 worms from nine individuals) and Ghana (46 worms from 11 individuals). Worms from different sites were collected using different protocols. During metagenomic analysis, we found that the storage of worms in RNAlater resulted in high viral read counts. Preliminary results identified a novel rhabdovirus. We are currently designing and validating a polymerase chain reaction (PCR) to be able to test serum samples of OAE patients for the presence of this virus. All other viruses were either from families not known to infect humans or sequenced in extremely low abundance.

In the current project, we propose to collect adult worms by nodulectomy and microfilariae by obtaining skin snips from persons with OAE and persons without epilepsy residing in an onchocerciasis-endemic area in South Sudan, and store them in RNAlater to optimise the yield. These will then be used for viral metagenomics analysis.

### Main study aim

To describe the *O. volvulus* virome and to identify viruses linked to onchocerciasis morbidity, specifically OAE.

### Study objectives

This study aims to collect, in an onchocerciasis endemic area in South Sudan, good quality samples of *O. volvulus* worms (microfilariae and adult forms). Collected samples will be analysed using viral metagenomics and investigated for the presence of the novel rhabdovirus identified in a pilot study in samples from Cameroon and Ghana.

Moreover, we will compare the number, size, and localisation of onchocerciasis nodules in persons with and without epilepsy. In addition, in the persons with epilepsy we will assess the effect of nodulectomy on the frequency of seizures.

### Hypothesis

The virome of *O. volvulus* worms obtained from persons with OAE differs from that obtained from persons without epilepsy. A novel rhabdovirus may also be found in *O. volvulus* samples from South Sudan.

## Methods

### Study design

An explorative case-control study.

### Study setting

**Figure 1.**
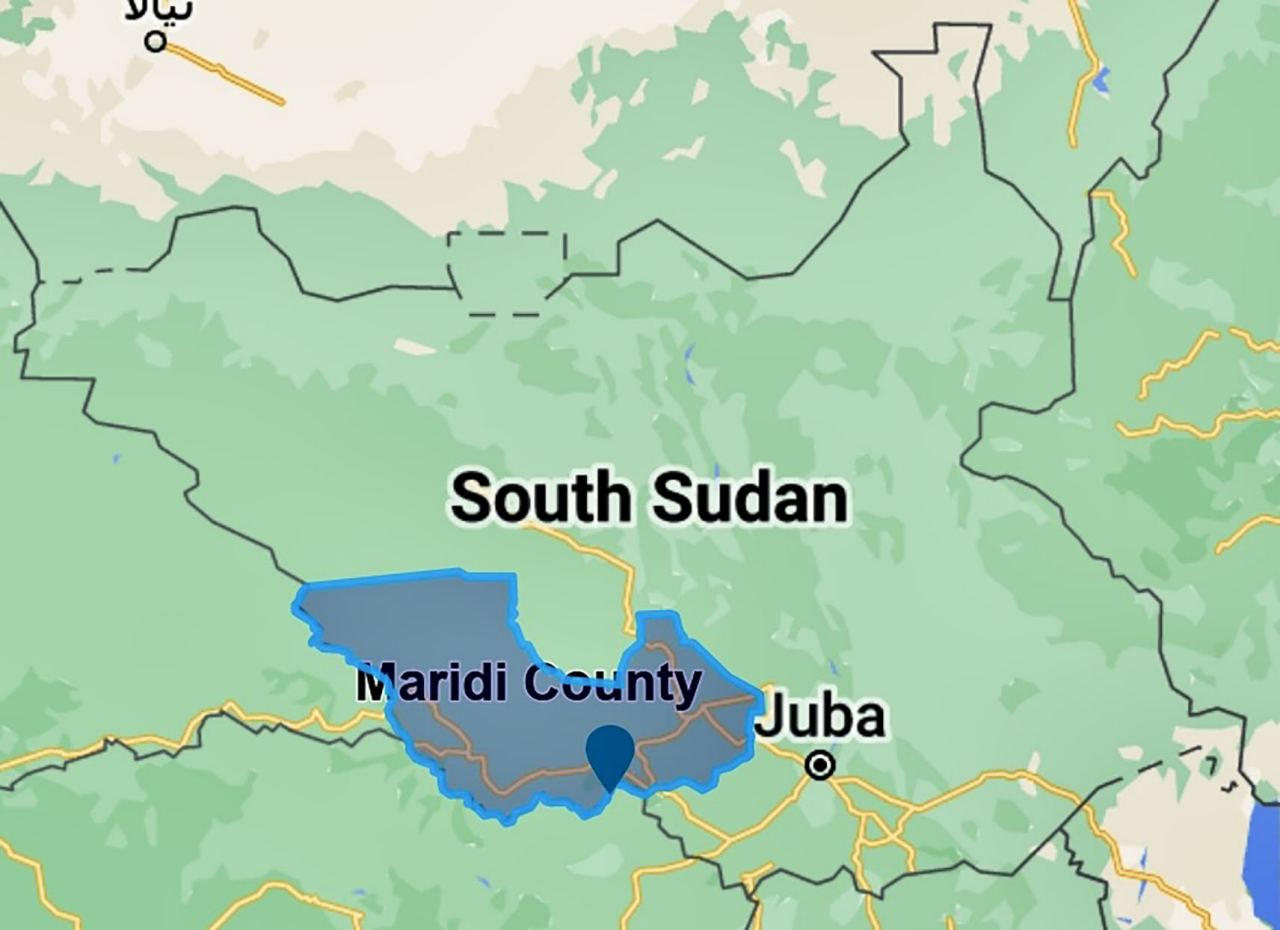
Location of Maridi County in Western Equatorian State, South Sudan (made in Scribble maps).

**Figure 2.**
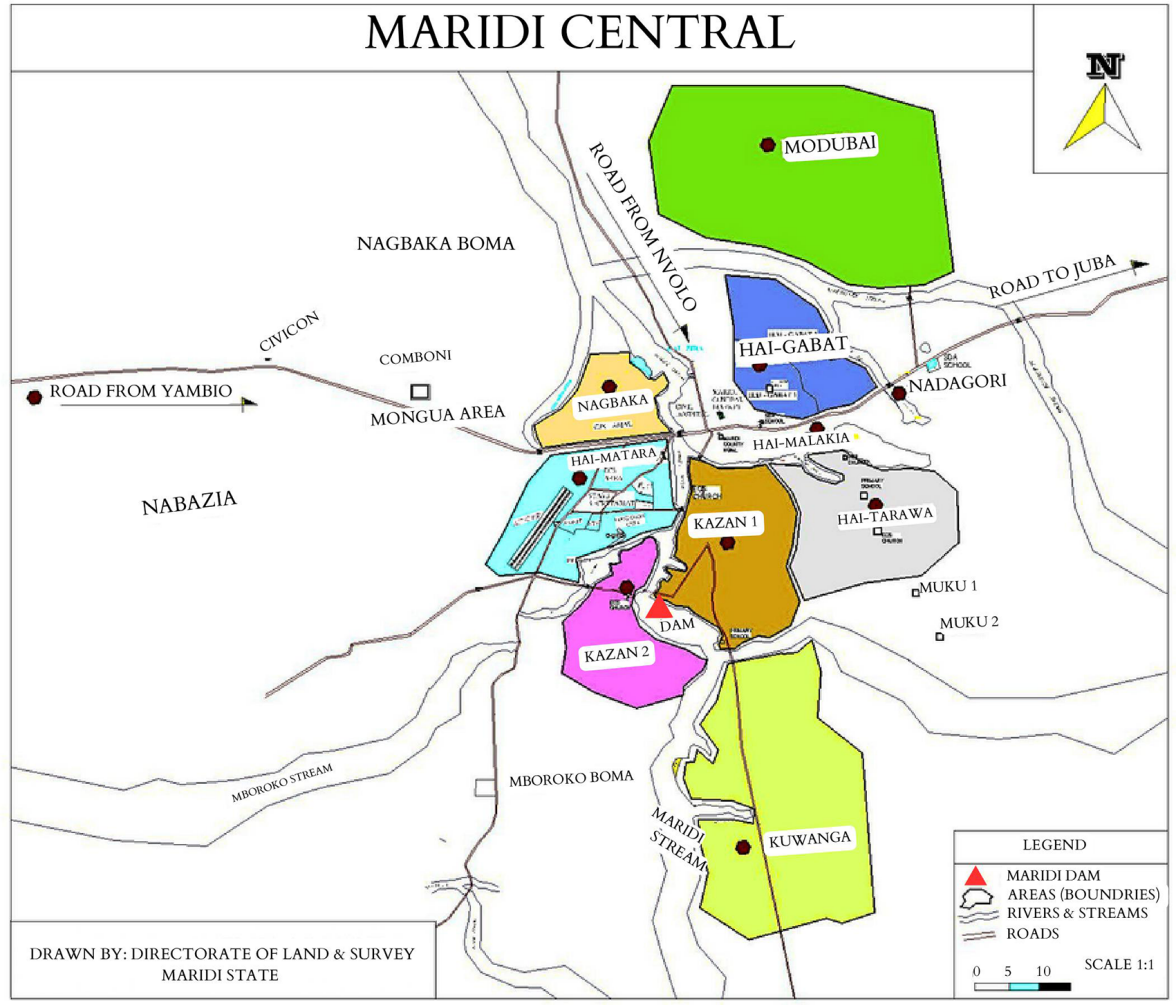
The geographical location of the eight study areas in Maridi County (adapted from Colebunders *et al.* ^16^).

South Sudan is known to have multiple endemic hotspots of onchocerciasis, including Maridi County in the Western Equatoria state ( Figure 1). Maridi County is home to over 115,000 individuals and is traversed by the Maridi River, upon which a dam was built in the 1950s. ^15^ The dam spillway was identified as the sole blackfly breeding site in Maridi, ^15^ fuelling onchocerciasis transmission in the neighbouring villages. In 2018, a high prevalence of epilepsy (4.4%) was documented in selected villages in Maridi, with a prevalence of 11.9% in Kazana-2, an area close to the Maridi Dam ( Figure 2). ^16^ Most (85.2%) of the persons in Maridi with epilepsy met the criteria of OAE in Maridi. ^17^

Annual community-directed treatment with ivermectin (CDTi) is the main method used for onchocerciasis control internationally and was introduced in Maridi in the early 2000s. However, there have been several years without treatment due to insecurity, which has allowed the disease to remain endemic in the area. The CDTi intervention was reintroduced in Maridi in 2017. Onchocerciasis transmission was assessed in December 2019 via an *O. volvulus* antibody serosurvey (using the OV16 rapid diagnostic test) in children aged 3 to 6 years and a seroprevalence of 40.0% was found in Kazana-2 village, suggesting very high ongoing onchocerciasis transmission. ^18^ Moreover, blackfly biting rates of 202 flies per man per hour were observed at the Maridi Dam. ^18^ Annual CDTi was interrupted in 2020 because of the COVID-19 pandemic but was continued biannually (six-monthly) in 2021. Additionally, in 2019, a community-based vector control strategy known as “Slash and Clear” was implemented at the Maridi Dam. This method consists of clearing the trailing vegetation at blackfly breeding sites to reduce biting density in the nearby communities. ^18^

These onchocerciasis elimination measures reduced the transmission of *O. volvulus* and OAE incidence in Maridi. ^19^ However, overall coverage of ivermectin of 80% is recommended to reach elimination of parasite transmission. ^20^ The CDTi coverage has been increasing but remains low in Maridi (only 56.6% of the population took ivermectin in 2021), and the onchocerciasis elimination goals remain far from being attained. ^19^

### Time schedule

The major milestones for this study will be spread over time as follows:

• Study preparation, including community sensitisation: August 2023
• Recruitment, including nodulectomies: September to December 2023
• Metagenomic analysis: January 2024

### Sample size calculations

Based on limited preliminary data, no sample size can be calculated. The 20 cases and 20 controls represent a convenience-based sample size and are proposed for reasons of feasibility and acceptability. The prevalence of onchocerciasis nodules in persons with and without OAE has so far not been well assessed. In a study concerning the clinical manifestations of epilepsy in Maridi, onchocerciasis nodules were only reported in 5.8% of persons with nodding syndrome and in 0.7% of persons with epilepsy without nodding syndrome. ^21^

### Study population and samples to be collected

Twenty OAE cases (≥12 years old) with at least one onchocerciasis nodule and 20 age- and village-matched non-epileptic controls with at least one onchocerciasis nodule will be recruited into the study. A person will be considered having OAE if he/she matches the criteria of the most recent case definition of OAE. ^5^

### Study procedures

Selection and enrolment of the study participants

After obtaining informed consent, persons with epilepsy attending the epilepsy clinic at Maridi Hospital, at least 12 years old and living in Kazana-1 or Kazana-2 villages, will be carefully examined for the presence of onchocerciasis nodules by trained personnel. The main parts of the body that will be examined are the head, neck, chest, arms, lumbar region, buttocks, thighs, and legs. Persons with epilepsy will also be interviewed to assess whether they meet the criteria of OAE. ^5^ If they meet these criteria, they will be asked to participate in the study. Recruitment of OAE cases will be consecutive.

After all persons with OAE have been selected for the study, controls will be identified during a house-to-house survey in Kazana-1 and Kazana-2. All persons without epilepsy between the ages of 12 and 30 years will be examined for the presence of onchocerciasis nodules. For each case with OAE, a control with at least one onchocerciasis nodule with a similar age (±3 years), sex and ivermectin use will be asked to participate in the study. Both OAE cases and controls will only be enrolled after written informed consent is obtained, as well as assent from the children 12–17 years old.

Interview and examination

For each study participant, pre-tested questionnaires ^22^ will be completed. This questionnaire will include questions about potential onchocerciasis-related symptoms (epilepsy, cognitive decline, skin, and eye problems) and past ivermectin use. All participants will be examined, and the number, size, and localisation of the nodules will be noted. Moreover, in follow-up questionnaires, ^23^ we will record the status of the wound and the number of seizures since the nodulectomy 10 days and one-month post-nodulectomy.

Nodulectomy

Extraction of adult worms by nodulectomy will be performed at Maridi Hospital by the project physician. Transport to the hospital and post-nodulectomy care will be organised by the research team. Nodulectomies will be performed under local anaesthesia and aseptic conditions by a medical doctor (TD) trained in the procedure. The skin above the nodule will be disinfected with 70% alcohol, followed by a povidone-iodine solution. Only superficially located nodules will be selected for nodulectomy. After extraction of the nodule, the skin wound will be sutured and covered by a band aid. Ten days after wound closure the wound will be inspected, and sutures removed.

Nodules will be incubated in collagenase (Gibco™; ThermoFisher; CNr° 17101015) to break up the outer layer. All worms will be washed thrice with 20% Percoll® (Sigma-Aldrich; PNr° P1644-25ML). All collected worms from one person will be pooled and submerged in RNAlater for storage.

Skin snip testing

One skin snip will be taken from either side of the iliac crest using a sclerocorneal biopsy punch. The skin snip will immediately be put into a 96-well microtitre plate with approximately 40 μL physiological saline. Biopsies will be incubated for 12–24h at room temperature. Afterwards, the emerged microfilariae will be counted using an inverted microscope (Leica DM IL LED; VWR CNr° 630-3462) and aspirated from the plate into a labelled tube for storage in RNAlater.

*O. volvulus* antibody testing

Blood will be obtained by finger prick for Ov16 rapid diagnostic testing. Moreover, four dry blood spots on filter paper and 5 mL of venous blood will be collected per person to allow for the possibility of using these samples for future pathogenesis and *O. volvulus* diagnostic studies.

Evaluation of seizures

In persons with epilepsy, the number of seizures will be assessed pre-nodulectomy and 10 days and one-month post-nodulectomy.

Storage and transfer of samples

The *O. volvulus* worms in RNAlater will be preserved initially at the Maridi hospital in a -20°C freezer. Later samples will be transferred under cold chain conditions, first to a -80°C freezer at the Public Health Laboratory of the Ministry of Health of South Sudan in Juba and later to a -80°C freezer at the Rega Institute at the KU Leuven in Belgium.

Viral metagenomic analysis

All collected worms will be sent to the Rega Institute under the Nagoya agreement. Worms will be processed following the NetoVIR protocol adapted for adequate homogenization. ^14^ In short, worms will be diluted in 500μl PBS and homogenized in a Precellys® Evolution tissue homogenizer (Bertin Technologies) with 2.8 mm zirconium oxide beads (Precellys) at 4500 rpm for 1 min. For each processed batch of worms, a negative control consisting of only PBS will be taken along. Next, the samples will be centrifuged at 17,000 g for 3 min and 150 μl supernatant of each sample will be subsequently filtered through a 0.8 μm filter (Sartorius). This filtrate will be treated with a mix of Benzonase (50 U, Novagen) and Micrococcal nuclease (2000 U, New England Biolabs) to digest remaining free-floating eukaryotic and bacterial nucleic acids. Viral DNA and RNA will be then extracted using the Kingfisher Flex system (Thermofischer), Applied Biosystems in combination with the MagMAX™ Viral/Pathogen Nucleic Acid Isolation Kit (Thermofischer). DNA and RNA will be amplified using the Complete Whole Transcriptome Amplification kit (WTA2, Merck), and resulting PCR products will be further purified and prepared for sequencing with the Nextera XT kit (Illumina). The final sequencing libraries will be cleaned up with Agencourt AMPure XP beads (Beckman Coulter, Inc.) using a 0.6X ratio of beads to sample. Finally, paired-end sequencing will be performed on the Nextseq 550 platform (Illumina) for 300 cycles (2x150bp) with an estimated 10 million reads per sample. All resulting sequences will be run through the ViPER pipeline ( https://github.com/Matthijnssenslab/ViPER) on the infrastructure of the Vlaamse (Flemish) Supercomputer Center® (VSC) to evaluate the read quality, perform *de novo* assembly, virus identification and classification to produce a first overview of virus species found in the obtained reads. Relevant viruses will be evaluated on their potential to infect humans and compared between cases and controls.

**Study outcomes and data analysis plan**

*O. volvulus* viromes will be examined for the potential presence of viruses related to families/clades able to infect humans, including the rhabdoviruses detected during the pilot study in samples from Cameroon and Ghana. In addition, the *O. volvulus* viromes of persons with and without OAE will be compared.

**Data handling and storage**

Research data management will be done according to the FAIR principles (research outputs are findable, accessible, interoperable, and reusable). All personal information will be encoded and treated confidentially with REDCap (Version: 13.7.3). Codified data will be entered into secure, password-protected spreadsheets and stored in a secured central server. All coded individual participant data underlying the results will be made available immediately and indefinitely via the Zenodo repository following publication for anyone who wishes to access the data for any purpose.

### Patient and Public Involvement

The study was prepared in collaboration with the Neglected Tropical Disease department of the Ministry of Health of the Republic of South Sudan. Prior to the study, communities will be sensitised and mobilised about the importance of preventing OAE by participating in the CDTi programme. The study rationale, objectives and procedures will be explained during interactive community meetings. Study findings will be communicated to the village communities as the project advances.

### Ethics and dissemination

The protocol has received ethical approval from the Ethics Committee of the University of Antwerp (Ref: 6 July 2023, B3002023000098) and of the Ministry of Health of South Sudan (Ref: 1 September 2023, MOH/RERB 45/2023). Written informed consent will be obtained from all participants and assent from the 12- to 17-year-old children. All collected data during the study will be treated confidentially. Only coded data relevant to the study will be recorded in a database. Findings will be disseminated nationally and internationally via meetings and peer-reviewed publications.

**Study status**

Enrolment of the first participants is planned for September 2023.

## Discussion

Recent epidemiological studies have shown that OAE is an important, still insufficiently recognised public health problem in many remote onchocerciasis endemic areas in sub-Saharan Africa with onchocerciasis elimination programmes that have been interrupted or work sub-optimally. ^1^ To address this public health problem, it is important to prove that onchocerciasis can induce epilepsy directly or indirectly. Strong epidemiological evidence for the association between onchocerciasis and epilepsy may not suffice to convince public health decision makers to take appropriate action, as solid proof of a causal relationship between onchocerciasis and epilepsy still needs to be established. Therefore, we proposed a viral metagenomic study of the *O. volvulus* worm to detect a potential unknown virus in the worm that could play a role in the pathogenesis of OAE. To do so, we will obtain microfilariae by skin snips and adult worms by nodulectomies from persons with and without epilepsy in Maridi County, South Sudan.

So far, little is known about the prevalence, number, size, and localisation of nodules in persons with and without OAE in onchocerciasis-endemic areas. In 2011, in an onchocerciasis-endemic area in west Uganda, Kaiser *et al.* observed a trend for both a higher proportion of nodule carriers (P = 0.065, Mantel–Haenszel chi-squared test) and a higher mean number of nodules per individual (P = 0.061, Kruskal–Wallis test) in persons with epilepsy than controls. ^24^ In a study in 2011, in the Mbam Valley, an onchocerciasis-endemic area in Cameroon, it was found that persons with epilepsy were two times more likely to present with a palpable nodule than controls (odds ratio = 2.5, 95% confidence interval = 1.24–5.36). ^25^ More recently published data suggest that the prevalence of onchocerciasis nodules in persons with OAE is low. ^21^^,^^26^ In a study in an onchocerciasis endemic region in Ituri in the Democratic Republic of Congo nodules were only reported in 3.7% of the person with epilepsy. ^26^ In Maridi in South Sudan nodules were palpated in 5.8% of persons with nodding syndrome and in 0.7% of persons with other forms of epilepsy. ^21^ However, it is important that the nodule prevalence among persons with OAE is reassessed in a study in which all participants are carefully examined by a person with onchocerciasis nodule palpation experience.

We do not expect that extraction of the adult worms by nodulectomy will significantly influence the frequency of seizures of persons with OAE during the short follow-up period. Prior to the introduction of ivermectin in the control of onchocerciasis, nodulectomy was used as the principal method of disease control in Mexico, Guatemala, and Ecuador. The widespread use of nodulectomy, particularly for the removal of head nodules, was associated with decreasing rates of blindness in Guatemala ^27^; and in Ecuador it was shown to reduce dermal and ocular microfilarial loads. ^28^^,^^29^ However, a study in Nigeria showed that in patients with established *O. volvulus* infections who remain exposed to reinfection, nodulectomy did not reduce microfilarial density to any significant degree. ^30^ So far, the effect of nodulectomy on the frequency of seizures in persons with OAE has never been evaluated. Monitoring the clinical and parasitological parameters of our study participants for a longer period (at least one year) may provide more insight about the impact of nodulectomy on human onchocerciasis.

To the best of our knowledge, this will be the first viral metagenomic study of *O. volvulus* worms from South Sudan. Given our preliminary findings of a novel rhabdovirus in *O. volvulus* worms from Ghana, this new study may provide new insights in the pathogenesis of OAE. Notwithstanding, a weakness of our study is that we will perform nodulectomies in persons with OAE who are at least 12 years old, meanwhile onset of OAE can be as early as three years of age. Therefore, the microfilariae we will obtain might not be the same that initially induced OAE. As such, we might not be able to detect a virus that might have induced epilepsy, except if the latter has persisted in the adult worms which can live for up to 10 years. Nonetheless, describing the virome of *O. volvulus* will provide us with invaluable information on the viruses present in this parasite. If we detect viruses that could potentially infect humans, we could design PCR tests to screen young children with and without epilepsy in onchocerciasis-endemic areas.

Another possibility would be that the virus, potentially playing a role in the pathogenesis of OAE, may be present in most or even all *O. volvulus* worms. Following this hypothesis, the event to induce epilepsy would be an introduction of the virus into brain facilitated by an increased permeability of the blood-brain barrier, for example, because of an inflammatory reaction during a systemic infection in a young child. ^6^ Considering this hypothesis, there would be no difference between OAE and non-OAE worm viromes.

This study will be the first to describe the number, size, and localisation of nodules in persons with and without OAE in an onchocerciasis-endemic area in South Sudan. However, given the short follow-up period (one month) it will be difficult to evaluate the effect of nodulectomy on the long-term frequency of seizures.

### Perspectives

Our study will increase our knowledge about the biology of *O. volvulus* and may lead to new insights into the pathogenesis of OAE. Additional studies should compare the *O. volvulus* viromes from South Sudan with the *O. volvulus* viromes from other countries. Moreover, viral metagenomic studies should be conducted in *Onchocerca ochengi* ^31^ and other filarial worms. Our project also has diagnostic and therapeutic potential since a virome specific to OAE could be used as a target for molecular tests or drugs. More knowledge about the pathogenesis of OAE could convince public health decision-makers and funders that the *O. volvulus* parasite is able to directly or indirectly cause epilepsy, and that onchocerciasis-endemic regions with a high prevalence of epilepsy need to be prioritised for strengthening onchocerciasis elimination programmes.

## Data Availability

No data are associated with this study. Zenodo: Questionnaire Onchocerciasis-associated epilepsy, an explorative case-control study with viral metagenomic analysis of Onchocerca volvulus, https://doi.org/10.5281/zenodo.8146410. ^22^ Zenodo: Follow up questionnaire Onchocerciasis-Associated Epilepsy, an explorative case-control study with viral metagenomic analysis of Onchocerca volvulus, https://doi.org/10.5281/zenodo.8334911. ^23^ Data are available under the terms of the Creative Commons Attribution 4.0 International license (CC-BY 4.0)
