# Supplementary material for: Onchocerciasis-associated epilepsy: an explorative case-control study with viral metagenomic analyses on   Onchocerca volvulus
Source: F1000Res. 2023 Dec 11;12:1262. Originally published 2023 Oct 4. [Version 2] doi: 10.12688/f1000research.138774.2 (PMC10911407; doi:10.12688/f1000research.138774.2)
Supplement: Peer review history

# Reviewer report: Reviewer response for version 2

**DOI:** 10.5256/f1000research.159821.r229106

**Published:** 2024-02-16

**Authors:** Jeffrey Loeb, Jesica Herric

**Affiliations:** 1 The University of Illinois, Chicago, Illinois, USA; 2 Medicine - Infectious Diseases, University of Illinois Chicago, Chicago, Illinois, USA

I think the authors have addressed my major concerns in this revised version.  I still think that limiting enrollment to only older kids will make it more difficult to identify new cases of epilepsy, but they are only planning on enrolling 20 subjects so this hurdle can probably be overcome.  I'm not sure that removing only one nodule could be expected to have any impact at all on seizures, especially with such a short follow-up time, but as this is only an exploratory aim this is probably ok.

Is the study design appropriate for the research question?

Yes

Is the rationale for, and objectives of, the study clearly described?

Yes

Are sufficient details of the methods provided to allow replication by others?

Yes

Are the datasets clearly presented in a useable and accessible format?

Yes

Reviewer Expertise:

Parasitology

We confirm that we have read this submission and believe that we have an appropriate level of expertise to confirm that it is of an acceptable scientific standard.

# Reviewer report: Reviewer response for version 2

**DOI:** 10.5256/f1000research.159821.r229105

**Published:** 2024-02-15

**Authors:** Sébastien Pion

**Affiliations:** 1 Université Montpellier, Montpellier, France; 2 UMI233, Institut de Recherce pour le Développement, Montpellier, France

I thank the investigators for considering and replying to my comments. I have no further comments to make and wish them the best. Looking forward to seeing their findings.

Is the study design appropriate for the research question?

Partly

Is the rationale for, and objectives of, the study clearly described?

Yes

Are sufficient details of the methods provided to allow replication by others?

Yes

Are the datasets clearly presented in a useable and accessible format?

Not applicable

Reviewer Expertise:

Parasitology, epidemiology

I confirm that I have read this submission and believe that I have an appropriate level of expertise to confirm that it is of an acceptable scientific standard.

# Reviewer report: Reviewer response for version 1

**DOI:** 10.5256/f1000research.152001.r222520

**Published:** 2023-11-30

**Authors:** Sébastien Pion

**Affiliations:** 1 Université Montpellier, Montpellier, France; 2 UMI233, Institut de Recherce pour le Développement, Montpellier, France

In this exploratory study, the investigators will compare the presence and diversity of viruses contained in *Onchocerca volvulus* worms.

 What is the use of *O. volvulus* antibody testing if all participants harbor onchocercal nodules? A positive test will confirm the obvious whereas a negative test would be quite confusing.

 There is at least one step missing in the protocol: from the nodulectomy to the worm processing: nodule digestion. This step usually takes hours and requires collagenase enzyme to act at a temperature of about 40°C. Can this step affect the survival of RNA viruses?

 Impact of nodulectomy on frequency of seizures: one can expect a short term impact if the presence of the nodule is directly involved in triggering the seizures, for example like cysticercosis nodules pressuring some parts of the brain. Here, the relationship between nodules and seizure is certainly indirect, and delayed. In addition, it is unlikely that a person harbor one single nodule. Most nodules are located in deeper tissues/organs. Therefore, for the above reasons, removing a single nodule will probably have no consequence on the frequency of seizures.

 It is unclear if microfilariae present in the skin snips will be screened for viruses too.

 If viruses are found after processing the worms, how to be sure that those viruses originate from the worms and not residual human tissues?

 Communities where OAE is observed are communities where onchocerciasis transmission is intense. If a pool of *O. volvulus* worms contain neurological impairing viruses is present in the community, they will be randomly transmitted to the whole population and those worms would be in competition to infect humans (or not) with *O. volvulus* population not containing the neurological viruses. Sampling one nodule per person is therefore subject to very unrepresentative results (and it is a well known limitation of examining one nodule and extrapolating during clinical trials for example).

Is the study design appropriate for the research question?

Partly

Is the rationale for, and objectives of, the study clearly described?

Yes

Are sufficient details of the methods provided to allow replication by others?

Yes

Are the datasets clearly presented in a useable and accessible format?

Not applicable

Reviewer Expertise:

Parasitology, epidemiology

I confirm that I have read this submission and believe that I have an appropriate level of expertise to confirm that it is of an acceptable scientific standard, however I have significant reservations, as outlined above.

# Response

**Published:** 2023-12-05

**Authors:** Robert Colebunders

**Affiliations:** Global Health Institute, University of Antwerp, Antwerp, Belgium

**Response to reviewer 2**

**Reviewer**

 In this exploratory study, the investigators will compare the presence and diversity of viruses contained in *Onchocerca volvulus* worms.

 What is the use of *O. volvulus* antibody testing if all participants harbour onchocercal nodules? A positive test will confirm the obvious whereas a negative test would be quite confusing.

**Response**

**The Ov16 test will be used to select persons with *O. volvulus* nodules, because certain nodules may not be caused by onchocerciasis but could be for example a cysticercosis cyst.**

**Text change:**

**à The following text will be replaced above “ Nodulectomy”**

*O. volvulus* antibody testing

 Blood will be obtained by finger prick for Ov16 rapid diagnostic testing as an additional prerequisite to verify the origin of the nodule. Moreover, four dry blood spots on filter paper and 5 mL of venous blood will be collected per person to allow for the possibility of using these samples for future pathogenesis and *O. volvulus* diagnostic studies.

**Reviewer**

 There is at least one step missing in the protocol: from the nodulectomy to the worm processing: nodule digestion. This step usually takes hours and requires collagenase enzyme to act at a temperature of about 40°C. Can this step affect the survival of RNA viruses?

**Response**

**We will perform nodule digestion **(“Nodules will be incubated in collagenase (Gibco™; ThermoFisher; CNr° 17101015) to break up the outer layer. **”) for a total of 9h in 0.5mg/ml collagenase in RPMI, at 37°C and 90RPM. We will check on the nodules every hour, and eventually tease away the human tissue and wash them trice to reduce contact time of the worm with the collagenase. As the worm has a sturdy cuticle, we expect the viruses inside of the worm to still be intact and the DNA/RNA will remain detectable.**

**Text change:**

 Nodules will be incubated in 0.5mg/ml collagenase (Gibco™; ThermoFisher; CNr° 17101015) for an expected total of 9h at 37°C and 90RPM to break up the outer layer. To reduce worm-collagenase contact time, nodules will be checked every hour until signs of digestion occur, to eventually manually tease away the human tissue. Subsequently, all worms will be washed thrice with 20% Percoll® (Sigma-Aldrich; PNr° P1644-25ML) to remove any remnants of collagenase and most potential human viral contaminants. All collected worms from one person will be pooled and submerged in RNAlater for storage.

**Reviewer**

 Impact of nodulectomy on frequency of seizures: one can expect a short-term impact if the presence of the nodule is directly involved in triggering the seizures, for example like cysticercosis nodules pressuring some parts of the brain. Here, the relationship between nodules and seizure is certainly indirect, and delayed. In addition, it is unlikely that a person harbor one single nodule. Most nodules are located in deeper tissues/organs. Therefore, for the above reasons, removing a single nodule will probably have no consequence on the frequency of seizures.

**Response**

**We agree that the likelihood that the nodulectomy will have an effect on the frequency of seizures is small but as we will follow-up the persons with epilepsy anyway at the Maridi clinic data on frequency of seizures will be available**.

**Reviewer**

 It is unclear if microfilariae present in the skin snips will be screened for viruses too.

**Response**

**Screening for viruses in skin snips is not planned in a first phase but will be considered at a later phase using PCR confirmation of the presence of viruses. Skin-snips are planned to determine the infection status and burden. As CDTI is given bi-annually in Maridi, South Sudan, we expect low positivity or negative skin snip results. This will provide us with additional data on individual ivermectin intake and the state of the worms we collected, as we don’t know the impact of ivermectin on the worm’s virome. Moreover, any additional metadata on clinical outcome of onchocerciasis, might reveal preliminary results on the effects of the virome on the infection dynamics.**

**Text change:**

 “To determine infection status, and the impact of ivermectin intake, one skin snip will be collected from either side of the iliac crest using a sclerocorneal biopsy punch.”

 “the identified viromes might not be the same that initially induced OAE.”

**Reviewer**

 If viruses are found after processing the worms, how to be sure that those viruses originate from the worms and not residual human tissues?

**Response**

**In a second phase, we plan to demonstrate the presence of the virus in the worms by immunofluorescence and electron microscopy. However, seeing that we will wash the worm thrice with 20% percoll, we aim to reduce the amount of human tissue contamination on the worm itself.**

 “Subsequently, all worms will be washed thrice with 20% Percoll® (Sigma-Aldrich; PNr° P1644-25ML) to remove any remnants of collagenase and most potential human viral contaminants.”

**Reviewer**

 Communities where OAE is observed are communities where onchocerciasis transmission is intense. If a pool of *O. volvulus* worms contain neurological impairing viruses is present in the community, they will be randomly transmitted to the whole population and those worms would be in competition to infect humans (or not) with *O. volvulus* population not containing the neurological viruses. Sampling one nodule per person is therefore subject to very unrepresentative results (and it is a well known limitation of examining one nodule and extrapolating during clinical trials for example).

**Response**

**We acknowledge that collecting one nodule per person might not be enough to represent the total exposure of the person, especially as we are collecting worms years after seizure onset. Though, we expect to collect multiple nodules from a single incision due to the occurrence of smaller ‘satellite’ nodules surrounding the ‘mother’ nodule. Nonetheless, we are in a very early stage of investigating the virome of *O. volvulus* and therefore any additional information will be valuable.**

**To learn more about the dynamics of the virome in this worm, we will perform metagenomics on single worms to identify intranodular variability. In addition we will investigate internodular variability if multiple nodules are recovered from the same person.**

**It is possible that this virus is present in all *O. volvulus* worms and is not directly associated with epilepsy. However, this study will provide a valuable amount of data on the virome of *O. volvulus* in the context of an OAE case-control study.**

**Text changes:**

 “Relevant viruses will be evaluated on their potential to infect humans and compared between cases and controls. Single worm viromes will be compared between and within patient nodules to identify inter- and intranodular virome variability to better understand the virome dynamics among worms.”

 “Only superficially located nodules will be selected for nodulectomy. All nodules accessable through a single incission will be extracted, as multiple smaller nodules are to be expected surrounding the larger ‘mother’ nodule. After extraction of the nodule(s), the skin wound will be sutured and covered by a band aid. Ten days after wound closure the wound will be inspected, and sutures removed.”

# Reviewer report: Reviewer response for version 1

**DOI:** 10.5256/f1000research.152001.r221394

**Published:** 2023-11-28

**Authors:** Jeffrey Loeb, Jesica Herric

**Affiliations:** 1 The University of Illinois, Chicago, Illinois, USA; 2 Medicine - Infectious Diseases, University of Illinois Chicago, Chicago, Illinois, USA

The authors present a very interesting plan for what could be a really landmark study - but we think that they need to make some refinements for the study to be robust and have meaningful results.

 The authors are investigating the virome of oncho-infected individuals to try and identify any viruses that could play a role in oncho-associated epilepsy. Importantly, the authors have identified a novel rhabdovirus present in oncho-infected individuals without epilepsy in two countries. They will also test individuals with epilepsy to see if they are infected with this virus (although the fact that those WITHOUT epilepsy are known to be infected with the virus means that even if it is present in those with epilepsy it will be tough to prove that the virus causes epilepsy). However, the overall goal of studying the virome in those with oncho with and without epilepsy is very important and will make for a very interesting study. The additional information they will collect regarding nodule distribution among these groups will also provide interesting information. 

 We're not sure why they have selected a population of individuals >12 years old to study when the peak incidence of oncho-associated epilepsy is 8-11 years. Unless there is a really good reason, they may want to change the study protocol to include kids >8 years old. 

 For evaluation of seizures, they say they will assess the number of seizures pre- and post-nodulectomy - it's not discussed, but we think the authors should also gather information about seizure semiology and see if this changes following nodulectomy as well. 

 Another weakness we foresee in the study design is that they will only remove one oncho nodule - this may not have a significant impact in the overall immunology or clinical condition of the host - why not remove all of the nodules (at least even in a small subset of patients)? Removing 1 of several nodules wouldn't completely answer the question of what impact removing nodules will have, as it still could be argued that any lack of changes seen could be due to the remaining nodules.

 Finally, we're not sure a one-month follow-up will really be sufficient to detect a difference in seizures. We think the authors should seriously consider extending this follow-up period, and if they do not, they need to justify this fully.

Is the study design appropriate for the research question?

Yes

Is the rationale for, and objectives of, the study clearly described?

Yes

Are sufficient details of the methods provided to allow replication by others?

Yes

Are the datasets clearly presented in a useable and accessible format?

Yes

Reviewer Expertise:

Parasitology

We confirm that we have read this submission and believe that we have an appropriate level of expertise to confirm that it is of an acceptable scientific standard, however we have significant reservations, as outlined above.

# Response

**Published:** 2023-11-29

**Authors:** Robert Colebunders

**Affiliations:** Global Health Institute, University of Antwerp, Antwerp, Belgium

We selected individuals >12 years old because nodulectomy is an invasive procedure and we prefer not to perform this procedure in small children without their consent. Children >12 years old will need to provide assent and their parents informed consent.

 We will document seizure semiology but there is currently no evidence that this will change after nodulectomy. Moreover the samples size is small and the persons with epilepsy are also treated with anti-seizure medication.

 We do not want to remove more than one nodule because nodulectomy is an invasive procedure. Investigating the clinical impact of the nodulectomy is only a secondary objective of the study.

 If there could be an effect of the nodulectomy on the frequency of seizures we expect this to be observed shortly after removal. After one month, all persons with epilepsy will continue to be followed at the epilepsy clinic in Maridi, where they receive anti-seizure medication, however not part of a study protocol.

# Response

**Published:** 2023-12-05

**Authors:** Robert Colebunders

**Affiliations:** Global Health Institute, University of Antwerp, Antwerp, Belgium

**Response to reviewer 1**

 The authors present a very interesting plan for what could be a really landmark study - but we think that they need to make some refinements for the study to be robust and have meaningful results.

 The authors are investigating the virome of oncho-infected individuals to try and identify any viruses that could play a role in oncho-associated epilepsy. Importantly, the authors have identified a novel rhabdovirus present in oncho-infected individuals without epilepsy in two countries. They will also test individuals with epilepsy to see if they are infected with this virus (although the fact that those WITHOUT epilepsy are known to be infected with the virus means that even if it is present in those with epilepsy it will be tough to prove that the virus causes epilepsy). However, the overall goal of studying the virome in those with oncho with and without epilepsy is very important and will make for a very interesting study. The additional information they will collect regarding nodule distribution among these groups will also provide interesting information. 

 We're not sure why they have selected a population of individuals >12 years old to study when the peak incidence of oncho-associated epilepsy is 8-11 years. Unless there is a really good reason, they may want to change the study protocol to include kids >8 years old. 

**Response**

**We selected individuals >12 years old because nodulectomy is an invasive procedure and we prefer not to perform this procedure in small children without their consent. Children >12 years old will need to provide assent and their parents informed consent.**

**Reviewer**

 For evaluation of seizures, they say they will assess the number of seizures pre- and post-nodulectomy - it's not discussed, but we think the authors should also gather information about seizure semiology and see if this changes following nodulectomy as well. 

**Response**

**We will document seizure semiology but there is currently no evidence that this will change after nodulectomy. Moreover, the samples size is small and the persons with epilepsy are also treated with anti-seizure medication.**

**Text change:**

 In persons with epilepsy, the number and types of seizures will be assessed pre-nodulectomy and 10 days and one-month post-nodulectomy.

**Reviewer**

 Another weakness we foresee in the study design is that they will only remove one oncho nodule - this may not have a significant impact in the overall immunology or clinical condition of the host - why not remove all of the nodules (at least even in a small subset of patients)? Removing 1 of several nodules wouldn't completely answer the question of what impact removing nodules will have, as it still could be argued that any lack of changes seen could be due to the remaining nodules.

**Response**

**We do not want to remove more than one nodule because nodulectomy is an invasive procedure. However, we do expect to collect multiple nodules per incision as most ‘mother nodules’ are surrounded by smalles ‘satallite’ nodules. Investigating the clinical impact of the nodulectomy is only a secondary objective of the study. Moreover all participants will receive ivermectin twice a year. Therefore, it is unlikely that removing additional nodules may have an added value.**

**Text change:**

 “Only superficially located nodules will be selected for nodulectomy. All nodules accessible through a single incision will be extracted, as multiple smaller nodules are to be expected surrounding the larger ‘mother’ nodule. After extraction of the nodule(s), the skin wound will be sutured and covered by a band aid. Ten days after wound closure the wound will be inspected, and sutures removed.”

**Reviewer**

 Finally, we're not sure a one-month follow-up will really be sufficient to detect a difference in seizures. We think the authors should seriously consider extending this follow-up period, and if they do not, they need to justify this fully.

**Response**

**If there could be an effect of the nodulectomy on the frequency of seizures, we expect this to be observed shortly after removal. After one month, all persons with epilepsy will continue to be followed up at the epilepsy clinic in Maridi, where they receive anti-seizure medication, however not as part of a study protocol.**
